# Risk factors for exposure of Baerveldt glaucoma drainage implants: a case-control study

**DOI:** 10.1186/s12886-020-01632-5

**Published:** 2020-09-10

**Authors:** Ayaka Edo, Koji Jian, Yoshiaki Kiuchi

**Affiliations:** Department of Ophthalmology and Visual Science, Graduate School of Biomedical Sciences, Hiroshima University, 1-2-3 Kasumi, Minami-ku, Hiroshima, 734-8551 Japan

**Keywords:** Glaucoma drainage device exposure, Baerveldt glaucoma drainage implant, Risk factor, Implant model

## Abstract

**Background:**

Glaucoma drainage implant exposure is one of the serious complications after glaucoma drainage implant surgery. The purpose of this study is to evaluate the risk factors for exposure of the device after implantation of a Baerveldt glaucoma drainage implant.

**Methods:**

This is a retrospective review of the medical records of all patients who underwent Baerveldt glaucoma drainage implant surgery at the Hiroshima University Hospital between April 1, 2012 and October 31, 2016, and who were followed for at least 6 months after surgery. We examined the risk factors for implant exposure based on data obtained from the medical records, with a particular focus on the differences in implant models.

**Results:**

A total of 80 eyes from 80 patients were identified; all patients were Japanese. In this study, the rate of Baerveldt glaucoma drainage implant exposure was 15.0% (12 of 80 eyes). The exposure rate for the BG 102-350 tended to be higher than that for the BG 101-350 and BG 103-250 (*p* = 0.092; adjusted odds ratio = 3.34; 95% confidence interval, 0.82–13.58). In the patients who had diabetic mellitus, the BG 102-350 showed a significant risk of implant exposure (*p* = 0.038; adjusted odds ratio = 15.36; 95% confidence interval, 1.17–202.59).

**Conclusions:**

In Baerveldt glaucoma drainage implant surgery in patients with diabetes, using the BG 102-350 was associated with greater risk of implant exposure compared with using the BG 101-350 or BG 103-250.

## Background

Reducing intraocular pressure (IOP) is a common method for suppressing the progression of glaucoma [1, 2]. If the condition cannot be controlled with eye drops, glaucoma surgery is required. One traditional form of glaucoma surgery is the trabeculectomy (TLE). Recently, glaucoma drainage device (GDD) implantation has become a popular glaucoma operation [3, 4]. A GDD is a filtration system consisting of a silicon tube and a plate. The tube is inserted into the eye, and the plate is fixed subconjunctivally. The aqueous humor is allowed to flow out of the eye through the tube and into the plate, and is absorbed into the surrounding tissue to reduce the IOP [2, 4]. The Baerveldt glaucoma drainage implant (BGI, Johnson & Johnson Vision, Santa Ana, CA, USA) is one of the most popular types of GDD [2, 5, 6].

GDD implantation can provide long-term IOP control that is at least as efficacious as TLE for glaucoma treatment [7, 8]. However, there are different complications between GDD implantation and TLE. GDD implantation has complications such as implant exposure, tube obstruction, diplopia and corneal edema [8–12]. Implant exposure is a particularly serious complication of GDD implantation, because exposure leads to significant risk for endophthalmitis [13, 14]. Although there have been several reports showing the risk factors for implant exposure, few studies have reported the risk for Asian people, despite the fact that some reports suggest that racial differences affect GDD exposure [11, 15–22].

There are three BGI models: the BG 101-350, BG 103-250 and BG 102-350. The BG 101-350 and BG 103-250 implants have straight tubes connected to each 350 mm^2^ and 250 mm^2^ plates. The BG 102-350 implant has a 5.3-mm^2^ plate called the Hoffman elbow, for inserting the tube from the pars plana into the vitreous cavity, in addition to the tube and plate. Here, we focused on the differences between BGI model types and examined the risks for implant exposure.

## Methods

We performed a retrospective review of the medical records of all patients who underwent BGI surgery at the Hiroshima University Hospital between April 1, 2012 and October 31, 2016, and who had been followed for at least 6 months after surgery. The patients who received more than two implants in one eye were excluded from the analysis. In the patients who had BGIs implanted into both eyes, the data from the right eye were used. Our study followed the principles of the Declaration of Helsinki, and was approved by the Institutional Review Board of Hiroshima University (#Rin 339-1).

All patients included in this study underwent BGI implantation. The surgical method was as follows. After incision of the conjunctiva, the tenon capsule was dissected and the two near rectus muscles were confirmed. An implant was fixed between the rectus muscles and 3-0 nylon was threaded through the tube. The tube was ligated with 8-0 Vicryl (Ethicon, Somerville, NJ, USA) and a Sherwood slit was made to prevent ocular hypertension. In general, we covered the tube and Hoffman elbow with a 6 × 6-mm half-thickness self-scleral flap. In eight severe cases, in which the conjunctival sac had been shortened, the tube and Hoffman elbow were covered with a human donor scleral graft. The plate was fixed 1-mm posterior to the origin of the rectus muscle, and the tube was inserted into: the anterior chamber; the posterior chamber, through the ciliary sulcus (1.5 mm from the limbus); or the vitreous cavity, through the pars plana (3.5 mm from the limbus).

The BG 101-350 or BG 103-250 was used in all patients without a history of pars plana vitrectomy. For those patients who had a history of pars plana vitrectomy or who needed pars plana vitrectomy, the BG 102-350 was used. The implant location was prioritized in the order of superior-temporal, inferior-temporal, superior-nasal and inferior-nasal.

The possible risk factors that were examined included patient age, sex, diabetes mellitus, type of glaucoma, intraocular surgeries prior to BGI implantation, implant model, implant location and tube entry site. The implant models were the BG 101-350, BG 103-250 and BG 102-350. The types of glaucoma included primary open-angle glaucoma, neovascular glaucoma, secondary glaucoma, pediatric glaucoma and chronic angle closure glaucoma. Secondary glaucoma included pseudoexfoliative glaucoma, inflammatory glaucoma and traumatic glaucoma. We determined patients’ history of diabetes based on self-report.

### Statistics

We classified the cases that required repair, as a result of BGI exposure, as the exposure group. Basic descriptive statistics were calculated and reported as mean ± standard deviation. Comparisons between the two groups were analyzed with t-tests for continuous covariates in univariate analysis and with Fisher’s exact test for discrete variables. Younger age, neovascular glaucoma, previous TLE and inferior implant location have previously been reported to be risk factors for GDD exposure [16, 17, 20–22]. As candidate risk factors, we selected five factors: age younger than 20 years old, neovascular glaucoma, BG 102-350 implantation, a history of 3 or more previous TLE operations and inferior implant location, based on the previous reports [16, 17, 20–22] about risk factors for GDD exposure and on the results of univariate analyses. Variables to be subjected to logistic regression analysis and Cox proportional hazard regression analysis were selected using a stepwise method. Subgroup analysis of diabetic patients was performed similarly. Calculations were performed using JMP pro 14.0.0 (SAS Institute Inc., Cary, NC, USA). *P* values of less than 0.05 were considered statistically significant.

## Results

Results from a total of 80 eyes from 80 patients were analyzed. Table 1 summarizes their demographic and clinical characteristics, and the results of univariate risk-factor assessment for BGI exposure. The mean follow-up period was 26.0 ± 15.0 months, and all patients were Japanese. The mean age was 57.0 ± 23.0 years-old and the subjects included 11 children under 20-years-old (13.8%).

**Table 1:** Demographic characteristics of patients who had a Baerveldt glaucoma drainage implant surgery

Characteristics	Exposure eyes (*n* = 12)	Control eyes (*n* = 68)	P value *
Age (years)			0.93
Mean ± SD	57.4 ± 19.8	56.8 ± 24.5	
Follow-up period (months)			0.94
Mean ± SD	26.2 ± 17.6	26.6 ± 14.5	
Sex			1.00
Male	7	38	
Female	5	30	
Eye			0.35
Right	9	40	
Left	3	28	
Diabetes mellitus			0.32
Yes	6	22	
No	6	46	
Type of glaucoma			0.51
Primary open angle	0	7	
Neovascular	6	20	
Secondary	5	28	
Pediatric	1	12	
Chronic angle closure	0	1	
Previous ocular surgery			1.00
Yes	12	63	
No	0	5	
History of ≥3 previous TLE, no. of eyes			0.46
Yes	4	15	
No	8	53	
Type of implant			0.099
BG 101-350/ BG 103-250	8	59	
BG 102-350	4	9	
Implant location			0.84
Superior-temporal	3	21	
Inferior-temporal	9	44	
Superior-nasal	0	1	
Inferior-nasal	0	2	
Implant location			0.74
Superior	3	22	
Inferior	9	46	
Tube entry site			0.50
Anterior chamber	3	24	
Posterior chamber	5	32	
Vitreous	4	12	
Type of patch graft			0.095
Self-sclera flap	9	63	
Human donor sclera	3	5	

SD: standard deviation, TLE: trabeculectomy, no.: number

* P values were obtained from two-sample t-tests for continuous variables and Fisher’s exact tests for categorical variables

Implant exposure occurred in 12 eyes (15.0%) out of the 80 eyes. The mean period to exposure was 13.42 ± 10.67 months (2–34 months). In the exposure cases, a BG 101-350 or BG 103-250 implant had been used in eight cases and a BG102-350 implant was used in four. Of the eight cases in which a BG 101-350 or BG 103-250 implant was exposed, seven were tube exposures and one was a plate exposure. In the four cases with BG 102-350 implant exposure, three cases had Hoffman elbow exposure and one had a plate exposure. Thus, in most of the exposure cases, the tube or Hoffman elbow was exposed (83.3%, 10 of 12). The two patients with plate exposure each had pars plana vitrectomy after BGI surgery.

The rate of exposure with the BG 102-350 was high; 4 of 13 (30.7%) among patients with that type of implant. In contrast to this, the BG 101-350 and BG 103–250 were exposed in 8 of 67 cases (11.9%). However, this difference did not reach statistical significance (*p* = 0.099).

Table 2 lists the risk factors for implant exposure that were selected by the stepwise method of logistic regression analysis. There was a trend to BG 102-350 having a higher risk of exposure, compared with BG 101-350 and BG 103-250 (*p* = 0.092; adjusted odds ratio (OR) = 3.34; 95% confidence interval (CI), 0.82–13.58), but it did not reach statistical significance.

**Table 2:** Risk-factors assessment for Baerveldt glaucoma drainage implant exposure

Risk factor	Adjusted odds ratio	95% confidence interval	*P* value *
Type of implant			
BG 102-350	3.34	0.82–13.58	0.092
BG 101-350/ BG 103-250	1.00		
History of ≥3 previous TLE, no. of eyes			
Yes	1.82	0.14–2.14	0.39
No	1.00		

TLE: trabeculectomy; no.: number

* Logistic regression analysis

In the exposure group, neovascular glaucoma was the most common diagnosis, at 50%, followed by secondary glaucoma (41.7%) and pediatric glaucoma (8.3%). In the control group, secondary glaucoma (41.2%) was the most common diagnosis and the remaining diagnoses consisted of neovascular glaucoma (29.4%), pediatric glaucoma (17.6%), primary open angle glaucoma (10.3%) and chronic angle closure glaucoma (1.5%). Because many patients (53 of 80, 66.3%) had previously undergone glaucoma surgery, in a superior location, more than half of the devices were implanted inferiorly (56 of 80, 68.8%).

Most of the patients who were implanted with a BG 102-350 had diabetes mellitus (9 of 13, 69.2%). Tables 3 and 4 show the results of subgroup analysis of the diabetic patients. Table 3 presents the characteristics of those patients and the resultant risk factors for BGI exposure in univariate analysis. Among the patients with diabetes, the exposure rate with BG 102-350 was 44.4% (4 of 9), whereas the exposure rate with a BG 101-350 or BG 103-250 was 10.5% (2 of 19). Table 4 shows the associated risk factors for implant exposure in patients with diabetes mellitus, as selected by logistic regression analysis. The BG 102-350 had a significantly greater risk for implant exposure (*p* = 0.038; adjusted OR = 15.36; 95% CI, 1.17–202.59). Using a BG 102-350 increased the risk of exposure to about 15 times the risk with BG 101-350 and BG 103-250 in the diabetic patients.

**Table 3:** Univariate analysis of the risk factors for Baerveldt glaucoma drainage implant exposure in diabetic patients

Characteristics	Exposure eyes (n = 6)	Control eyes (*n* = 22)	P value *
Age (years)			0.49
Mean ± SD	60.3 ± 12.3	64.6 ± 12.2	
Follow-up period (months)			0.22
Mean ± SD	36.3 ± 15.1	26.4 ± 15.0	
Sex			1.00
Male	4	13	
Female	2	9	
Eye			1.00
Right	4	14	
Left	2	8	
Type of glaucoma			0.23
Primary open angle	0	3	
Neovascular	4	17	
Secondary	2	2	
Previous ocular surgery			1.00
Yes	5	18	
No	1	4	
History of ≥3 previous TLE, no. of eyes			0.14
Yes	3	4	
No	3	18	
Type of implant			0.064
BG 101-350/ BG 103-250	2	17	
BG 102-350	4	5	
Implant location			1.00
Superior-temporal	2	7	
Inferior-temporal	4	14	
Superior-nasal	0	1	
Implant location			1.00
Superior	2	8	
Inferior	4	14	
Tube entry site			0.42
Anterior chamber	0	3	
Posterior chamber	2	11	
Vitreous	4	8	

SD: standard deviation, TLE: trabeculectomy, no.: number

* P values were obtained from two-sample t-tests for continuous variables and Fisher’s exact tests for categorical variables

**Table 4:** Multivariate analysis of the risk factors for Baerveldt glaucoma drainage implant exposure in the diabetic patients

Risk factor	Adjusted odds ratio	95% confidence interval	*P* value *
Type of implant			
BG 102-350	15.36	1.17–202.59	0.038 **
BG 101-350/ BG 103-250	1.00		
History of ≥3 previous TLE, no. of eyes			
Yes	12.41	0.82–185.96	0.068
No	1.00		
Implant location			
Inferior	0.32	0.027–3.78	0.36
Superior	1.00		

TLE: trabeculectomy; no.: number

* Logistic regression analysis

** Statistically significant, *P* < 0.05

Cox proportional hazards regression model analysis for implant exposure in the diabetic patients is shown in Table 5. In such analyses, the use of the BG 102-350, history of 3 or more previous TLE and inferior implant location did not show a significant risk for implant exposure (BG 102-350: *p* = 0.061, OR = 5.52, 95% CI 0.92–46.99; 3 or more previous TLE: *p* = 0.062, OR = 6.01, 95% CI 0.90–49.00; inferior implant location: *p* = 0.28, OR = 0.34, 95% CI 0.05–2.79).

**Table 5:** Cox proportional hazard regression analysis for Baerveldt glaucoma drainage implant exposure in the diabetic patients

Risk factor	Odds ratio	95% confidence interval	P value *
Type of implant			
BG 102-350	5.52	0.92–46.99	0.061
BG 101-350/ BG 103-250	1.00		
History of ≥3 previous TLE, no. of eyes			
Yes	6.01	0.90–49.00	0.062
No	1.00		
Implant location			
Inferior	0.34	0.05–2.79	0.28
Superior	1.00		

TLE: trabeculectomy; no.: number

* Cox proportional hazard regression analysis

## Discussion

In our study, we examined exposure rates and risk factors for exposure after BGI implantation. The rate of exposure for BGI implanted at Hiroshima University Hospital between April 1, 2012 and October 31, 2016 was 15.0%. This rate is higher than the 5–6% rate that has been reported previous studies [8, 19, 20]. We consider that the causes of the high exposure rates in our study are related to following three things.

First, we consider that racial differences may have affected. Whereas previous reports have mainly focused on Caucasians, our reports have focused on Asians. Asians might have more friction of the GDD against the ocular tissues than do Caucasians. Friction between the ocular tissue and the GDD may increase the risk for GDD exposure. The average palpebral-fissure width among Asians is narrower than that in Caucasians [23]. Muir et al. also pointed out small orbits as the reason for the high exposure risk in children and women [19]. We speculate that Asians with tight orbits may have more friction with the implant than Caucasians, leading to increased exposure. Moreover, the prevalence of dry eye disease is higher in Asians than in Caucasians, which may increase the friction on the ocular surface and lead to GDD exposure [24].

Second, most of the patients in our study had had prior ocular surgeries before GDD implantation. In some studies, prior surgeries have been reported to be a risk factor for exposure [16, 21]. In Byun et al.’s study, the majority of patients had no history of prior ocular surgeries (153 out of 256, 57.5%) [16]. In Levinson et al.’s study, 18.2% of included cases had not had previous ocular surgery [20]. However, in our study, only 6.1% of patients had not.

Third, the use of different patch-graft materials to cover the tubes may have affected the rate of implant exposure. In our facility, due to the limited number of human donor scleral grafts, we covered the tubes with a self-scleral flap in most cases. Human donor scleral grafts were used only in the eight most-severe cases, in which the conjunctival sac had been shortened. However, previous studies about implant exposure in Westerners have mostly used donor sclera, cornea or pericardium [19, 20]. A previous report showed that the tube-exposure rate was 30% when the surgeons initially covered the tube with a self-scleral graft, but that the exposure rate was lowered to less than 5% with the advent of donor or autologous tissue for covering the tube [25]. The BGI exposure rates in Asians has only been reported by Tojo et al. [26]. They counted only tube exposure in their report, and the tube exposure rate was 3.8%. In their study, they used human donor scleral grafts in all cases, and nearly half of the cases were robustly covered with donor scleral graft and self-scleral flap. Perhaps, Asians’ eyes need to be covered with more robust patch graft to prevent exposure than those of Caucasians.

We observed that use of the BG 102-350 in diabetic patients was associated with 15-times greater odds of BGI exposure, compared with the use of BG 101-350 and BG 103-250. Of the four cases in which the BG 102-350 was exposed, the Hoffman elbow exposure occurred in three cases, except for one case in which plate was exposed after pars plana vitrectomy for diabetic retinopathy. Unlike the BG 101-350 and BG 103-250, the BG 102-350 has a Hoffman elbow, which is a 5.3-mm^2^ plastic plate. The Hoffman elbow is larger and harder than the tube. The exposure of a Hoffman elbow always started from the tip of the wing. Because a Hoffman elbow moves up and down, with the scleral insertion part acting as a fulcrum, the force to lift the tip of the Hoffman elbow’s wing might easily be applied by eye movements and blinking. It is conceivable that friction between the Hoffman elbow and ocular tissue, including the conjunctiva, is likely to occur. Moreover, it is well known that diabetes delays wound healing [27]. Owen et al. reported that there is poor conjunctival perfusion in diabetic patients and that it may result in poor tissue strength [28]. Because diabetic patients may have weaker tissue strength due to poorer conjunctival blood flow, in diabetic patients implant exposure might occur more easily with the BG 102-350 with its Hoffman elbow.

We consider that implantation of BG 101-350 or BG 103-250 would be a better way to prevent exposure in diabetic patients. When anterior type of implant such as BG 101-350 or BG 103-250 is inserted into the posterior chamber or vitreous cavity, the tube may touch the lens or the backside of the iris [29]. Depending on the patient’s condition, if BG 102-350 implantation is required, it will need to be covered with a more robust patch graft.

Our study has some limitations. First, it is limited by the retrospective analysis. Because the information was not uniformly recorded for each patient, there were several factors including the history of ocular surface disease and systemic disease other than diabetes that we could not examine, which might have had an association with implant exposure. Second, it included a small sample size, particularly in terms of examining the risk factors for implant exposure. The small sample size may have made it difficult to detect significant risk factors for exposure. We found that the BG 102-350 had a higher risk of exposure than other BGI types in patients with diabetes. However, it is unclear whether the BG 102-350 also represents a risk of exposure in non-diabetic patients. In our study, we were not able to analyze the relationship between BG 102-350 and exposure in non-diabetic patients since the BG 102-350 was implanted in only a few non-diabetic patients. Moreover, we were unable to show that the BG 102-350 was a significant risk factor in diabetic patients in the Cox proportional hazards model, unlike the results from logistic regression analysis. This might be due to the present research being a retrospective study with a small sample size. Although we have shown that the BG 102-350 could be involved in implant exposure in diabetic patients, this needs to be verified in future prospective studies with larger sample sizes and longer follow-up periods.

## Conclusions

Since the exposure of BGI increases the risk for endophthalmitis, exposure is one of the most-notable complications in BGI surgery. This study demonstrated that using the BG 102-350 is a risk for implant exposure in diabetic patients. We suggest that this should be investigated with a larger study population in the future, and that especially in the diabetic patients, it may require modifying the implant selection and surgical procedures to minimize the exposure risk.

## Data Availability

The datasets used and analyzed during the current study are available from the corresponding author on reasonable request.

## Abbreviations

BGI: Baerveldt glaucoma drainage implant
IOP: Intraocular pressure
GDD: Glaucoma drainage device
TLE: trabeculectomy
OR: odds ratio
CI: confidence interval

## References

[1] Heijl A, Leske C, Bengtsson B, Hyman L, Bengtsson B, Hussein M (2002). Reduction of intraocular pressure and Glaucoma progression. Arch Ophthalmol.

[2] Christakis PG, Zhang D, Budenz DL, Barton K, Tsai JC, Ahmed IIK (2017). Five year pooled data analysis of the Ahmed Baerveldt comparison study and the Ahmed versus Baerveldt study. Am J Ophthalmol.

[3] Minckler DS, Francis BA, Hodapp EA, Jampel HD, Lin SC, Samples JR (2008). Aqueous shunts in glaucoma: a report by the American Academy of ophthalmology. Ophthalmology..

[4] Arora KS, Robin AL, Corcoran KJ, Corcoran SL, Ramulu PY (2015). Use of various Glaucoma surgeries and procedures in Medicare beneficiaries from 1994 to 2012. Ophthalmology..

[5] Christakis PG, Tsai JC, Zurakowski D, Kalenak JW, Cantor LB, Ahmed II (2011). The Ahmed versus Baerveldt study: design, baseline patient characteristics, and intraoperative complications. Ophthalmology..

[6] Wang S, Gao X, Qian N (2016). The Ahmed shunt versus the Baerveldt shunt for refractory glaucoma: a meta-analysis. BMC Ophthalmol.

[7] Gedde SJ, Schiffman JC, Feuer WJ, Herndon LW, Brandt JD, Budenz DL (2012). Treatment outcomes in the tube versus trabeculectomy (TVT) study after five years of follow-up. Am J Ophthalmol.

[8] Gedde SJ, Herndon LW, Brandt JD, Budenz DL, Feuer WJ, Schiffman JC (2012). Postoperative complications in the tube versus trabeculectomy (TVT) study during five years of follow-up. Am J Ophthalmol.

[9] Krishna R, Godfrey DG, Budenz DL, Escalona-Camaaño E, Gedde SJ, Greenfield DS (2001). Intermediate-term outcomes of 350-mm2 Baerveldt glaucoma implants. Ophthalmology..

[10] Oana S, Vila J (2012). Tube Exposure Repair. J Curr Glaucoma Pract.

[11] Geffen N, Buys YM, Smith M, Anraku A, Alasbali T, Rachmiel R (2014). Conjunctival complications related to Ahmed Glaucoma valve insertion. J Glaucoma.

[12] Budenz DL, Feuer WJ, Barton K, Schiffman J, Costa VP, Godfrey DG (2016). Postoperative complications in the Ahmed Baerveldt comparison study during five years of follow-up. Am J Ophthalmol.

[13] Gedde SJ, Scott IU, Tabandeh H, Luu KK, Budenz DL, Greenfield DS (2001). Late endophthalmitis associated with glaucoma drainage implants. Ophthalmology..

[14] Alhadlaq A, Almalki S, Alshahwan S (2015). Late onset endophthalmitis associated with unexposed glaucoma valved drainage device. Saudi Journal of Ophthalmology.

[15] Morad Y, Donaldson CE, Kim YM, Abdolell M, Levin AV (2003). The Ahmed drainage implant in the treatment of pediatric glaucoma. Am J Ophthalmol.

[16] Byun YS, Lee NY, Park CK (2009). Risk factors of implant exposure outside the conjunctiva after Ahmed glaucoma valve implantation. Jpn J Ophthalmol.

[17] Koval MS, El Sayyad FF, Bell NP, Chuang AZ, Lee DA, Hypes SM (2013). Risk factors for tube shunt exposure: a matched case-control study. J Ophthalmol.

[18] Huddleston SM, Feldman RM, Budenz DL, Bell NP, Lee DA, Chuang AZ (2013). Aqueous shunt exposure: a retrospective review of repair outcome. J Glaucoma.

[19] Muir KW, Lim A, Stinnett S, Kuo A, Tseng H, Walsh MM (2014). Risk factors for exposure of glaucoma drainage devices: a retrospective observational study. BMJ Open.

[20] Levinson JD, Giangiacomo AL, Beck AD, Pruett PB, Superak HM, Lynn MJ (2015). Glaucoma drainage devices: risk of exposure and infection. Am J Ophthalmol.

[21] Trubnik V, Zangalli C, Moster MR, Chia T, Ali M, Martinez P (2015). Evaluation of risk factors for Glaucoma drainage device-related erosions: a retrospective case-control study. J Glaucoma.

[22] Netland P, Chaku M, Ishida K, Rhee D. Risk factors for tube exposure as a late complication of glaucoma drainage implant surgery. Clin Ophthalmol. 2016:547–53. 10.2147/OPTH.S104029 PMC4820194 27099461

[23] Viveiros MMH, Matai O, Takahagi RU, Padovani CR, Schellini SA (2017). Eyelid fissure dimensions in Japanese and in Brazilians of European descent over 50 years of age. Arq Bras Oftalmol.

[24] Albietz JM, Lenton LM, McLennan SG (2005). Dry eye after LASIK: comparison of outcomes for Asian and Caucasian eyes. Clin Exp Optom.

[25] Heuer DK, Budenz D, Coleman A (2001). Aqueous shunt tube erosion. J Glaucoma.

[26] Tojo N, Hayashi A, Consolvo-Ueda T, Yanagisawa S (2018). Baerveldt surgery outcomes: anterior chamber insertion versus vitreous cavity insertion. Graefes Arch Clin Exp Ophthalmol.

[27] Greenhalgh DG (2003). Wound healing and diabetes mellitus. Clin Plast Surg.

[28] Owen CG, Newsom RSB, Rudnicka AR, Ellis TJ, Woodward EG (2005). Vascular response of the bulbar conjunctiva to diabetes and elevated blood pressure. Ophthalmology..

[29] Tojo N, Ueda-Consolvo T, Yanagisawa S, Hayashi A (2017). Baerveldt® glaucoma implant surgery with the double scleral flap technique to prevent Hoffman elbow exposure. Graefes Arch Clin Exp Ophthalmol.

